# Red Ginger Extract Prevents the Development of Oxaliplatin-Induced Neuropathic Pain by Inhibiting the Spinal Noradrenergic System in Mice

**DOI:** 10.3390/biomedicines11020432

**Published:** 2023-02-02

**Authors:** Keun-Tae Park, Heejoon Jo, Bonglee Kim, Woojin Kim

**Affiliations:** 1 Department of Physiology, College of Korean Medicine, Kyung Hee University, Seoul 02453, Republic of Korea; 2 Korean Medicine-Based Drug Repositioning Cancer Research Center, College of Korean Medicine, Kyung Hee University, Seoul 02447, Republic of Korea

**Keywords:** noradrenaline, oxaliplatin-induced neuropathic pain, red ginseng

## Abstract

Oxaliplatin is a well-known chemotherapeutic drug that is widely used to treat colorectal cancer. However, it can induce acute side effects in up to 90% of patients. Serotonin and norepinephrine reuptake inhibitors (SNRIs) are used as first-choice drugs; however, even SNRIs are known to be effective only in treatment and not for prevention. Therefore, finding a drug that can prevent the development of cold and mechanical forms of allodynia induced by oxaliplatin is needed. This study demonstrated that multiple oral administrations of 100 mg/kg and 300 mg/kg of red ginger extract could significantly prevent pain development in mice. The role of the noradrenergic system was investigated as an underlying mechanism of action. Both the spinal α1- and α2-adrenergic receptors were significantly downregulated after treatment. Furthermore, the noradrenaline levels in the serum and spinal cord were upregulated and downregulated after treatment with paclitaxel and red ginger, respectively. As the active sub-component of red ginger, ginsenoside Rg3 (Rg3) was identified and quantified using HPLC. Moreover, multiple intraperitoneal injections of Rg3 prevented the development of pain in paclitaxel-treated mice, suggesting that RG3 may induce the effect of red ginger extract.

## 1. Introduction

Oxaliplatin, a platinum derivative, is a chemotherapeutic agent commonly used to treat colorectal, gastric, and pancreatic cancers worldwide [1,2]. In the treatment of these cancers, oxaliplatin has shown clinically significant advantages in the adjuvant setting and for treating metastatic disease [3]. However, although patient survival has greatly increased owing to oxaliplatin treatment, it causes several side effects that reduce the quality of life [4]. The main side effect is neurotoxicity, which manifests as acute peripheral sensory neuropathy and can be accompanied by significant neuronal damage [5]. The acute cold and mechanical forms of allodynia are some of the most severe complications of oxaliplatin treatment, as it decreases patient quality of life and even leads to chemotherapy discontinuation. Oxaliplatin-induced peripheral neuropathy (OIPN) persists in some cases; the incidence range of chronic OIPN is 64–97%, and 12% of patients experience severe neuropathy. Clinical data have shown that more than 60% of patients reduce or discontinue oxaliplatin owing to these side effects, affecting their chances of survival [6].

Although many studies have been conducted to find a peripheral neuropathy prevention method, an effective treatment has yet to be found [7]. According to the clinical practice guidelines established by the American Society of Clinical Oncology in 2014, no drugs are recommended to prevent this disease [8].

Panax ginseng has been used worldwide for thousands of years to treat cancer [9], diabetes [10], and postmenopausal symptoms [11]. Korean red ginseng is steam-treated Panax ginseng, which suggests that the chemical transformation of active compounds involves ginsenosides, peptides, and polysaccharides [12]. The less polar ginsenosides Rg3, Rg5, F4, and Rk1 are unique constituents of red ginseng [13,14,15,16]. The effects of red ginseng extract (RGE) have been reported in various studies. In clinical trials, it has been reported to treat acute respiratory illnesses [17] and cancer-related fatigue [18], increased postoperative immunity in gastric cancer patients [19], and decreased stress [20]. Animal studies have shown that the antioxidant effect alleviates type I diabetes [21] in rodents. In addition, ginsenosides are known to be the major components of RGE. Several studies have demonstrated powerful biological activities, such as vasodilation, neuroprotection, and radical scavenging [22,23,24,25]. In particular, ginsenoside Rg3 (Rg3) was shown to inhibit the stress-induced hypothalamus pituitary adrenal response by increasing NO production in the brain [26], protect cultured rat cortical cells from glutamate-induced neurodegeneration [27], and antagonize platelet-activating factor [28].

This study aimed to assess the preventive effects of multiple oral treatments with RGE on oxaliplatin-induced cold and mechanical forms of allodynia. Furthermore, to understand the underlying mechanism of RGE, the study aimed to estimate the noradrenaline levels in the serum and spinal cord and the two spinal adrenergic receptors. Finally, to understand the role of Rg3 in the preventive effect of RGE, the study aimed to investigate the effects of three different doses of Rg3 on oxaliplatin-induced neuropathic pain and to quantify the Rg3 content in RGE by HPLC. To the best of our knowledge, this is the first study to report that RGE potently prevents pain in a mouse model of oxaliplatin-induced peripheral pain.

## 2. Materials and Methods

### 2.1. Collection and Preparation of Red Ginseng Extract (RGE)

The red ginseng used in this experiment was produced by KOREA INSAM Co., Ltd. (Wonju, Republic of Korea). The red ginseng was extracted using a reflux method with 55% ethanol for 16 h at 85 °C. The extracts were filtered and concentrated under decompression and at 60 °C. The content of the red ginseng used in the experiments has been well reported by several papers [29,30,31] and the content of ginsenoside Rg3 in the red ginseng extract used in the experiment was identified by HPLC. The RGE was diluted in PBS to a concentration of 10 mg/mL. Furthermore, 100 mg/kg or 300 mg/kg of RGE was orally administered to the mice.

### 2.2. Animals

Six-week-old male C57BL/6 mice purchased from Daehan Biolink (Chungbuk, Republic of Korea) were used in these experiments. Mice were acclimated for 1 week under standard conditions (23 ± 2 °C, 65 ± 5% humidity, 12 h light/12 h dark cycles). The animals were maintained on a standard diet (Purina, Sungnam, Republic of Korea) with freely available water. The mice were housed and used in strict accordance with the guidelines established by the Kyung-Hee University Animal Care and Use Committee (approval no.: KHUASP-20-448).

### 2.3. Oxaliplatin, REG, and Rg3 Administration

Oxaliplatin (6 mg/kg, Sigma Aldrich, St. Louis, MO, USA) was dissolved in 5% glucose at a concentration of 2 mg/mL depending on a weight to intraperitoneal injection of 0.1 mL. The vehicle control group was administered the same volume of 5% glucose via the same route. The RGE was orally administered to the mice at concentrations of 100 and 300 mg/kg, and the Rg3 was injected intraperitoneally at concentrations of 0.05, 0.5, 5, and 50 mg/kg. RGE and Rg3 were administered once daily for 5 days (D-2–D2), and oxaliplatin was administered on the third day (D0). The Rg3 was first dissolved in 10% DMSO and further diluted in PBS according to the administered dose.

### 2.4. Behavior Tests

Behavioral tests were performed to examine the different sensory components of neuropathic pain before and after oxaliplatin administration. The cold and mechanical forms of allodynia were measured using acetone drop and von Frey filament tests, respectively. Cold allodynia was measured using the acetone test [32]. The mice were placed in a plastic cage with a wire mesh floor and allowed to habituate for 30 min before testing. The acetone drop test was used to measure the responses to an innocuous cold stimulus, and an acetone drop (10 µL) was sprayed onto the plantar skin of each hind paw three times. Latency to respond and withdrawal frequency for 30 s after acetone spraying.

**Figure 1:**
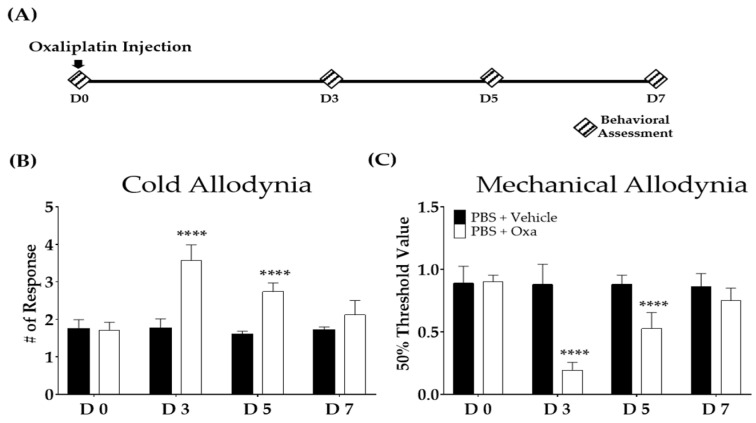
The effect of a single intraperitoneal oxaliplatin injection in mice. Schedule for experiments conducted to assess the degree of the cold and mechanical forms allodynia after oxaliplatin injection (**A**). Cold (**B**) and mechanical (**C**) allodynia after oxaliplatin injection. *N* = 6 each group. Note: **** *p* < 0.0001 vs. vehicle with two-way ANOVA followed by Sidak’s post-test for multiple comparisons. The ANOVA test found interaction *F*-values of 32.8 and 28.1 for the cold and mechanical forms of allodynia, respectively.

Mechanical allodynia was measured using the von Frey filament test [33]. A von Frey filament with a bending force of 0.02–2 g was applied to the mid-plantar skin of each hind paw for a maximum of 5 s or until licking, flinching, or paw withdrawal occurred. The A 50% threshold was calculated based on Dixon’s up–down and Chaplan’s calculation methods [34]. The pain was confirmed when a significant difference in the behavior was shown after cold and mechanical stimuli between the vehicle- (5% glucose) and oxaliplatin-injected mice (Figure 1).

### 2.5. Tissues Collection

The mice used in the qPCR and HPLC analyses were anesthetized via the inhalation of isoflurane until they were unresponsive. The mice were sacrificed by transcardial perfusion under isoflurane anesthesia. All tissues were stored at −80 °C until analysis.

### 2.6. Quantitative Real-Time Polymerase Chain Reaction (RT-PCR)

The total RNA from the spinal cord was extracted using the AccuPrep RNA Extraction Kit (Bioneer, Daejeon, Republic of Korea), according to the manufacturer’s protocol. The RNA concentration was quantified using a NanoDrop ND-1000 spectrophotometer (Thermo Scientific, Middlesex Country, MA, USA). The cDNA was prepared using Maxime RT Premix (Intronbi, Seongnam, Republic of Korea). The quantitative reverse transcriptase polymerase chain reaction (qRT-PCR) testing was performed using a SensiFAST SYBR No-ROX kit (Bioline, London, UK) and a CFX Real-Time PCR System (Bio-Rad, Hercules, CA, USA). The oligonucleotide primers used for the PCR were as follows: α1-adrenergic receptor (*Adra1a*) forward 5′-ATG CTC CAG CCA AGA GTT CA-3′ and reverse 5′-TCC AAG AAG AGC TGG CCT TC-3′; α2-adrenergic receptor (*Adra2a*) forward 5′-AAA CCT CTT CCT GGT GTC TC-3′; *Gapdh* forward 5′-GGA GGT AGC TCC TGA TTC GC-3′ and reverse 5′-CAC ATT GGG GGT AGG AAC AC-3′. The reaction was conducted under conditions of preheating for 10 min at 95 °C, followed by 40 cycles of 95 °C for 20 s, 57 °C for 20 s, and 72 °C for 20 s. The PCR data were quantified based on the number of cycles required for the amplification-generated fluorescence to reach a specific threshold of detection (Ct value). The relative gene expression was quantified based on the average Ct value of each gene for equal amounts of RNA (0.5 μg) and the average Ct value for each gene. The normalized expression change was recorded as 2^−ΔΔCt^ (*Gapdh* control was set to 1).

### 2.7. Noradrenaline Analysis in the Serum and Spinal Cord

The blood samples were collected via cardiac puncture under isoflurane anesthesia. The serum samples were separated via centrifugation at 10,000× *g* for 5 min at 4 °C. All serum samples were stored at −80 °C until analysis. The spinal cord was homogenized in 0.5 mL PBS. After centrifugation at 10,000× *g* for 10 min at 4 °C, the supernatant was collected. The soluble protein concentration of the supernatant was determined using a Bio-Rad protein assay (Hercules, CA, USA) with a BSA standard. The noradrenaline levels in the serum and spinal cord were measured using an enzyme-linked immunosorbent assay (ELISA) (Abnova, Walnut, CA, USA) according to the manufacturer’s instructions.

### 2.8. Identification and Quantification of Ginsenosides in RGE

The phytochemical compound of the RGE was injected and analyzed using an Agilent 1260 Infinity II HPLC and UV detector. The conditions for the ginsenoside standard mix and the Rg3 analysis are listed in Table 1. A stock solution of Rg3 and ginsenoside in a standard mix (100 µg/mL) was prepared in methanol. The Rg3 and ginsenoside standard mix used in this study was purchased from Sigma-Aldrich (St. Louis, MO, USA). Five dilutions of Rg3 (100, 50, 25, 12.5, 6.25 ug/mL) were prepared and subjected to an HPLC analysis. The standard solution was stored at −20 °C in the dark until analysis. Here, 100 mg of the sample was ultrasonically extracted (4 °C, 20 min) using 1 mL of 50% methanol. The diluted solution was centrifuged (4 °C, 10,000× *g* rpm, 5 min), and the supernatant was filtered through a 0.45 µm syringe filter to obtain a test solution.

## 3. Results

### 3.1. Oxaliplatin Injection Induces Cold and Mechanical Forms of Allodynia in Mice

The cold and mechanical forms of allodynia were induced in mice after intraperitoneal injection of oxaliplatin. The acetone drop and von Frey tests were used to the assess cold and mechanical forms of allodynia, respectively. Behavioral tests were conducted on the first (D0), third (D3), fifth (D5), and seventh (D7) days following the initial injection (Figure 1A) of oxaliplatin. In the oxaliplatin-injected group, the responses to cold (Figure 1B) and mechanical (Figure 1C) stimuli significantly increased from D3 to D5 compared to the control group. However, on D7, the pain was no longer significantly different between the two groups.

### 3.2. Anti-Allodynic Effect of RGE

**Figure 2:**
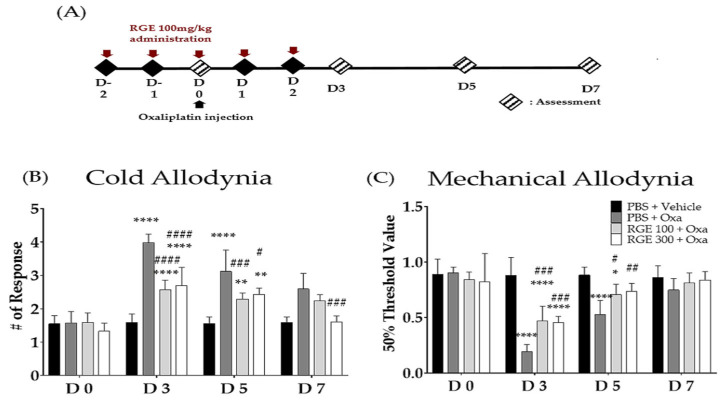
Effect of red ginseng extract (RGE) 100, 300 mg/kg on the cold and mechanical forms of allodynia induced by oxaliplatin injection in mice. The experimental schedule (**A**) and the effects of RGE against oxaliplatin-induced cold (**B**) and mechanical (**C**) forms of allodynia in mice. Behavioral tests to measure allodynia were performed before (D0) and after three times a day (D3, D5, D7). PBS and vehicle (5% glucose) were used as controls as they are solvents of RGE and oxaliplatin, respectively. *N* = 6 for each group; **** *p*, ^####^*p* < 0.0001, ^###^ *p* < 0.001, ** *p,* ^##^ *p* < 0.005, * *p,* ^#^ *p* < 0.05 vs. vehicle group with a two-way ANOVA followed by Sidak’s post-test for multiple comparisons. The ANOVA test found interaction *F*-values of 12.42 and 8.966 for the cold and mechanical forms of allodynia, respectively.

We estimated the preventive effects of RGE against oxaliplatin-induced neuropathic pain. Compared with the vehicle group, the oxaliplatin-administered group induced the cold and mechanical forms of allodynia significantly. The pretreatment with RGE (300 mg/kg, i.p.) once a day for five consecutive days (Figure 2A) significantly decreased the cold and mechanical forms of allodynia from days 3 to 5 (Figure 2B,C).

### 3.3. Involvement of Spinal Noradrenergic Receptors in the Analgesic Effect of RGE

**Figure 3:**
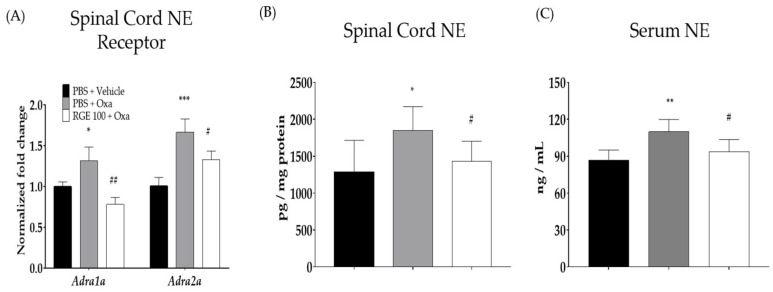
Quantitative RT-PCR testing of adrenergic receptors in the spinal cord and noradrenaline levels in the serum and spinal cord. The effects of oxaliplatin on spinal *α*1- and *α*2-adrenergic receptors (**A**) and noradrenaline levels in the serum (**B**) and spinal cord (**C**). Note: *α*1-ADR: *α*1-adrenergic receptor; *α*2-ADR: *α*2-adrenergic receptor; NE: noradrenaline. *N* = 6 for each group. Data were experiments and expressed as mean value ± SD. Note: *** *p* < 0.001, ** *p*, ^##^ *p* < 0.01, * *p*, ^#^ *p* < 0.05 vs. control with Student’s *t*-test.

The gene expression of noradrenergic receptors was evaluated to understand the underlying mode of action of red-ginseng-induced analgesia. The qRT-PCR testing was performed to determine the effects of oxaliplatin and RGE (100 mg/kg) on spinal α1- and α2-adrenergic receptors on day 3. When measuring the expression of α1-noradrenergic receptors in the spinal cord, the value of the vehicle group was established as 1 ± 0.15, and the oxaliplatin-induced group significantly increased to 1.31 ± 0.16. The group treated with oxaliplatin and RGE significantly decreased to 0.78 ± 0.08 (Figure 3A). The expression of the α2-noradrenergic receptor in the vehicle group was 1 ± 0.10, while that in the oxaliplatin-induced group significantly increased to 1.66 ± 0.16. However, in the RGE group, it significantly decreased to 1.33 ± 0.10.

### 3.4. Noradrenaline Levels in the Spinal Cord and Serum

The effects of oxaliplatin and RGE on neuropathic pain in mice were examined by measuring the noradrenaline levels in the spinal cord and serum. When measuring the noradrenaline levels in the spinal cord, the values for the vehicle group, oxaliplatin-induced group, and RGE-administered group were 1279.7 ± 436.2, 1848.1 ± 323.6, and 433.7 ± 269.6 pg/mg protein, respectively, and both increases and decreases were significant (Figure 3B). An analysis of the noradrenaline levels in the serum also showed very similar results. As a result of the noradrenaline levels in the serum, the result for the vehicle group was 86.7 ± 8.3 ng/mL, while the oxaliplatin-induced group significantly increased to 109.8 ± 10.1 ng/mL. The group treated with oxaliplatin and RGE significantly decreased to 93.6 ± 9.9 ng/mL (Figure 3C).

### 3.5. Identification and Quantification of Ginsenosides in RGE

**Figure 4:**
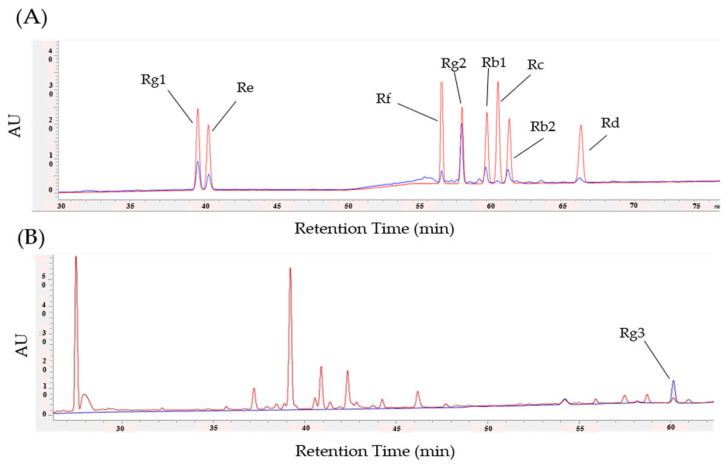
Identification and quantification of ginsenosides in RGE using HPLC. HPLC chromatograms of Rg1, Rg2, Re, Rf, Rb1, Rb2, Rc, Rd (**A**), and Rg3 (**B**). The peak in blue represents the standard and the red line represents the ginsenosides. The *X*-axis reports the retention time (RT) and the *Y*-axis reports the absorbance unit (AU).

The HPLC was conducted to identify and quantify major ginsenosides components such as Rg1, Rg2, Rg3, Re, Rf, Rb1, Rb2, Rc, and Rd in the RGE (Figure 4). The retention time (RT) and spectrum (AU) values of the standards and Rg1, Rg2, Re, Rf, Rb1, Rb2, Rc, Rd (Figure 4A), and Rg3 (Figure 4B) solutions were consistent. The calibration curve shows the linearity of the detector over the Rg3 range (100, 50, 25, 12.5, 6.25 μg/mL). The Rg3 regression equations were y = 3.91996x + 4.53153 and RSQ = 0.99976, and the content of Rg3 RGE was approximately 0.052% (Figure 4B). The quantification results for each ginsenosides are presented in Table 2.

### 3.6. Anti-Allodynic Effect of Rg3

**Figure 5:**
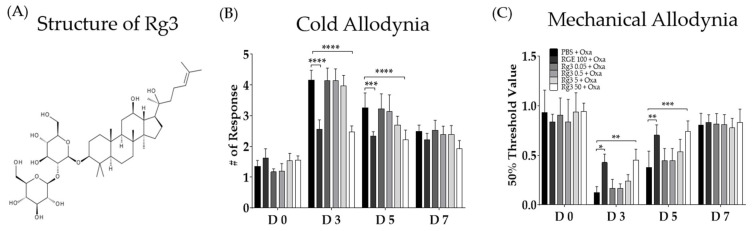
Dose-dependent preventive effects of ginsenoside Rg3 on the cold and mechanical forms allodynia induced by oxaliplatin injection in mice. The chemical structure of ginsenoside Rg3 (**A**). Effects of ginsenoside Rg3 against oxaliplatin-induced cold (**B**) and mechanical (**C**) forms of allodynia in mice. Behavioral tests to measure allodynia were performed before (D0) and three, five, and seven days after (D3, D5, D7) a single oxaliplatin injection. PBS and vehicle were used as the controls as they are solvents of both RGE and Rg3.P *N* = 6 for each group; **** *p* < 0.0001, *** *p* < 0.001, ** *p* < 0.01, * *p* < 0.05 vs. vehicle group with two-way ANOVA followed by Sidak’s post-test for multiple comparisons. The ANOVA test found interaction *F*-values of 8.966 and 2.771 for the cold and mechanical forms allodynia, respectively.

Although numerous ginsenosides are present in RGE, we further focused on Rg3, as its role in oxaliplatin-induced neuropathic pain has never been elucidated. The preventive effect of Rg3 on the development of allodynia after the oxaliplatin injection was assessed (Figure 5). Behavioral tests were conducted after the RGE and Rg3 pretreatments in oxaliplatin-induced neuropathic pain mice. To elucidate whether Rg3 could dose-dependently block the oxaliplatin-induced allodynia, four different doses (0.05, 0.5, 5, and 50 mg/kg) of Rg3 were administered intraperitoneally. The results showed that only the highest dose of Rg3 (i.e., 50 mg/kg) could prevent the oxaliplatin-induced cold (Figure 5C) and mechanical (Figure 5D) forms of allodynia. The effect of Rg3 was similar to that of 100 mg/kg of RGE.

## 4. Discussion

Oxaliplatin-dependent neuropathy is an ongoing challenge in cancer treatment, as it can significantly affect the quality of life and survival of cancer patients [35]. According to the published guidelines of the American Society of Clinical Oncology (ASCO), no agents are recommended for prevention, and serotonin-noradrenaline reuptake inhibitor (SNRIs) are recommended for the treatment of oxaliplatin-induced neuropathic pain [36]. SNRIs increase the action of serotonin and noradrenaline and are involved in endogenous analgesic mechanisms [37,38]. One SNRI, venlafaxine, has been reported to be effective in neuropathic pain, such as in polyneuropathy and diabetic neuropathy in humans [39,40]. However, compared with many clinical reports, studies on the prevention and mechanism of neuropathic pain induced by chemotherapeutic agents are still insufficient [41,42].

In this study, 100 mg/kg and 300 mg/kg of RGE significantly prevented the development of pain in oxaliplatin-treated mice. Although both doses of RGE were effective, we conducted further studies with 100 mg/kg of RGE, as its effect was stronger on days 3 and 5 (Figure 2) when the pain was strongly induced after oxaliplatin injection (Figure 1). Furthermore, the noradrenergic system was revealed to be associated with the analgesic action of red ginseng, as the spinal α_1_- and α_2_-noradrenergic receptors and the amounts of noradrenaline in the spinal cord and serum increased after the cisplatin injection and decreased following the red ginseng extract treatment. Repeated treatments with RGE inhibited the progression of oxaliplatin-induced neuropathy, which was evaluated as a decrease in the pain threshold or an increase in suprathreshold stimulation. The pain relief effect gradually increased during the treatment, suggesting a mechanism of pain suppression through the induction of pain by hormones and neurotransmitters.

The content of Rg3, which is expected to be an active ingredient of RGE and a key component in pain relief, was analyzed using HPLC (Figure 4B). The amount of Rg3 contained in the RGE was 0.057%, and when 100 mg/kg of RGE was ingested, the administered Rg3 was approximately 0.05 mg/kg. In this study, there was a difference between the preventive effects of 100 mg/kg of RGE and 0.05 mg/kg of Rg3. Although a preventive effect of Rg3 at 50 mg/kg was shown, its content differed from the content of Rg3 contained in the RGE at 100 mg/kg. It is expected that RGE is a complex extract, and it is expected that Rg3 interacts with unknown substances to show a preventive effect. Furthermore, although their effects were not assessed in this study, Rg1 and Rg2 and may also have played an important role, as they are the two major components (i.e., 0.89% and 1.84% of the RGE, respectively) in the RGE among the eight ginsenosides analyzed. The effects of Rg1 and Rg2 against oxaliplatin-induced neuropathic pain have never been assessed; however, for paclitaxel-induced neuropathic pain, multiple intravenous injections of 5 and 10 mg/kg of Rg1 significantly alleviated the allodynia and sciatic nerve TNF-α pain [43]. The effect of Rg2 was assessed in a chronic constriction injury of the sciatic nerve (CCI)-induced neuropathic pain mice, and multiple intraperitoneal injections of 5 and 10 mg/kg of Rg2 significantly increased the lowered threshold to mechanical and thermal stimuli [44]. Moreover, both Rg1 and Rg2 were shown to modulate the macrophages to decrease the production of pro-inflammatory cytokines (i.e., TNF-α, IL-1β, and IFN-β), as Rg1 inhibited the chemotherapy-induced microglial polarization from the M2 to M1 phenotypes [45] and Rg2 inhibited the toll-like receptor 4 (TLR4)-mediated signaling pathways in peritoneal macrophages [46]. However, future studies should be conducted to confirm their preventive or treatment effects against oxaliplatin-induced neuropathic pain.

The analysis of noradrenaline receptor gene expression in the spinal cords of mice demonstrated the involvement of the spinal noradrenergic (NE) system as a mechanism of action. In our study, both spinal α1- and α2-adrenergic receptors were shown to be increased after oxaliplatin treatment and decreased after 100 mg/kg of RGE treatment. Several studies have associated an increase in spinal noradrenergic receptors with pain.

The oxaliplatin injection increased the gene expression of spinal α1- and α2-adrenergic receptors and increased the NE levels in the serum and spinal cord. The importance of α1- and α2-adrenergic receptors in neuropathic pain has been widely reported [47,48]. The administration of α1- and α2-adrenergic receptor antagonists to mice with neuropathic pain induced by nerve damage was demonstrated to significantly attenuate the pain, indicating that both receptors are involved in pain increases [49]. The RGE (100 and 300 mg/kg) and Rg3 (50 mg/kg) were administered, the oxaliplatin-induced neuropathic pain was significantly attenuated, and the noradrenaline levels in the serum and spinal cord were significantly reduced.

Rg3 is a tetracyclic triterpenoid saponin. Recently, the neuroprotective effects of Rg3 have been extensively studied [50,51]. In addition, intraperitoneal injections of Rg3 for 4 weeks suppressed trimethyltin-induced seizures, improved the behavioral function by regulating the PI3K/AKT signaling pathway, and exhibited neuroprotective effects against focal cerebral ischemia in mice [25,52].

In conclusion, our results show that oxaliplatin-induced neuropathic pain can be considered a preventive agent because RGE and Rg3 significantly attenuated pain. However, the optimal therapeutic doses of RGE and Rg3 for neuropathic pain have yet to be fully elucidated. A major limitation of the current study is that the exact mechanisms of RGE against neuropathic pain have not been identified and require further research. In addition, it is necessary to further investigate the constituents of RGE and search for unknown substances exhibiting preventive effects except such as Rg3.

## Figures and Tables

**Table 1:** Analytical conditions of HPLC for the ginsenosides analysis.

	Conditions					
Treatment	Ginsenoside Rg3			Ginsenoside standard mix (Rg1, Rg2, Re, Rf, Rb1, Rb2, Rc, Rd)		
Column	Fortis extended-C18 (4.6 mm × 250 mm, 5 μm)			Fortis extended-C18 (4.6 mm × 250 mm, 5 μm)		
Flow rate	1 mL/min			1 mL/min		
Injection volume	10 μL			10 μL		
UV detection	203 nm			203 nm		
Run time	75 min			105 min		
Gradient	Time	%DW	%ACN	Time	%DW	%ACN
	0	80	20	0	80	20
	5	80	20	25	80	20
	20	77	23	45	75	25
	25	70	30	50	65	35
	45	60	40	75	60	40
	55	50	50	80	95	5
	65	50	50	90	95	5
	70	80	20	95	20	80
	75	80	20	105	20	80

**Table 2:** Quantified ginsenoside contents in the RGE.

	Ginsenoside Analysis								
	Rg1	Rg2	Rg3	Rb1	Rb2	Rc	Rd	Re	Rf
Contents (%)	0.89	1.84	0.05	0.48	0.50	0.06	0.19	0.54	0.19
Retention time (min)	39.630	57.492	60.173	59.130	60.646	59.905	65.495	40.396	56.087

## Data Availability

The data presented in this study are available on request from the corresponding author.
